# Diagnostic accuracy of NMP 22 and urine cytology for detection of transitional cell carcinoma urinary bladder taking cystoscopy as gold standard

**DOI:** 10.12669/pjms.36.4.1638

**Published:** 2020

**Authors:** Muhammad Tanveer Sajid, Muhammad Rafiq Zafar, Hussain Ahmad, Saif Ullah, Zahoor Iqbal Mirza, Khubaib Shahzad

**Affiliations:** 1 Dr. Muhammad Tanveer Sajid, FCPS. Assistant Professor, Armed Forces Institute of Urology (AFIU), Rawalpindi, Pakistan; 2 Prof. Muhammad Rafiq Zafar, FCPS. Associate Professor, Armed Forces Institute of Urology (AFIU), Rawalpindi, Pakistan; 3 Dr. Hussain Ahmad, FCPS. Associate Professor, Armed Forces Institute of Urology (AFIU), Rawalpindi, Pakistan; 4 Dr. Saif Ullah, Armed Forces Institute of Urology (AFIU), Rawalpindi, Pakistan; 5 Prof. Zahoor Iqbal Mirza, FCPS. Armed Forces Institute of Urology (AFIU), Rawalpindi, Pakistan; 6 Dr. Khubaib Shahzad, FCPS. Associate Professor, Armed Forces Institute of Urology (AFIU), Rawalpindi, Pakistan

**Keywords:** Cystoscopy, Nuclear matrix protein 22, Nuclear matrix-associated proteins, Sensitivity, Specificity, Transitional cell carcinoma, Urinary bladder neoplasms, Urine cytology

## Abstract

**Objective:**

To determine diagnostic accuracy of NMP 22 and urine cytology in the detection of transitional cell carcinoma (TCC) urinary bladder taking cystoscopy as a gold standard in patients having provisional diagnosis of bladder cancer (BC).

**Methods:**

This cross sectional validational study enrolled 380 patients fulfilling selection criteria and was conducted at Armed Forces Institute of Urology (AFIU) Rawalpindi, Pakistan form July 2018 to July 2019. The urine sample collected underwent NMP22 and cytological analysis followed by rigid cystoscopy. Reports of all three tests divided patients into positive or negative for malignancy as per defined criteria. Sensitivity, specificity, positive predictive value (PPV), negative predictive value (NPV) and diagnostic accuracy of NMP 22, urine cytology and their combination was determined. Receiver operating characteristic (ROC) curve analysis performed and area under the curve (AUC) compared among these tests.

**Results:**

The average age of patients was 53.08 ± 12.41 years having male to female ratio 3.75:1(300 males and 80 females). NMP 22 had better sensitivity and comparable specificity to cytology (81.9 & 81.2% vs 54 & 93.9%). Combination of NMP 22 / cytology outperformed both in terms of sensitivity (91.63 vs 81.83 vs 53.96), NPV (87.59 vs 77.46 vs 61.02) and diagnostic accuracy (85.26 vs 81.58 vs 71.32) but at the cost of specificity (76.97 vs 81.21 vs 93.94) and PPV (83.83 vs 85.02 vs 92.06). ROC curve revealed statistically significant higher AUC (0.843 vs .815 vs .73) for combination as compared to NMP 22 and Cytology (p < 0.001).

**Conclusion:**

NMP22 is a quick, point of care test having higher sensitivity, NPV and accuracy but similar specificity and PPV to urine cytology for detection of TCC urinary bladder. Combination outperformed both in terms of sensitivity while having modest specificity.

## INTRODUCTION

Transitional cell carcinoma (TCC) urinary bladder is one of the most common as well as lethal urological cancer worldwide accounting for about 95% of bladder cancers (BC).^1^ An estimated 81,190 new cases of BC with 17,240 deaths occurred in United States in 2018 and disease is emerging as global menace due to steadily rising life expectancy vis-a-vis heavily incurred health care expenditure (£55 million per year to investigate hematuria in UK).^2^ BC incidence and mortality is highest amongst developed nations but lowest in Asia, Latin America and the Caribbean; the variegated geographical distribution being attributed to global variation in risk factors mainly industrialization and smoking exposure.^3^

BC carcinogenesis revolves around genetic susceptibility, environmental exposure and unhealthy lifestyles.^4^ Majority are non-muscle invasive (NMIBC) having high chances of recurrence as well as progression necessitating lifelong surveillance.^5^ Comprehensive, standardized and risk-adapted follow-up protocols incorporating regular cystoscopies, urinary cytology as well as regular upper urinary tract imaging (for high-risk tumors) are recommended by various international bodies like European Association of Urology (EAU), the American Urological association (AUA), society of urological oncology and National comprehensive cancer network (NCCN).^6^ In addition to diagnosis, surveillance of BC recurrence pivots cystoscopy as criterion standard due to its high diagnostic accuracy. However, it is invasive, expensive and resource intensive compounded by low patient acceptance as well as poor compliance.^7^ Urine cytology is another commonly used gold standard modality for BC screening having excellent specificity. The drawbacks include low sensitivity especially for low grade tumors, requirement of trained cytopathologist and the impossibility of quantification due to subjective interpretation criteria.^8^ These compelling limitations lead to approval of several noninvasive biomarkers though with conflicting diagnostic accuracy quoted for primary and recurrent BC.^9^

Nuclear matrix protein (NMP), first described in 1974, is a nonchromatin structure that supports nuclear shape and organizes DNA having up to 25 times higher urinary levels in BC patients. The NMP-22 BladderChek test is an in vitro enzyme immunoassay intended for the qualitative detection of nuclear mitotic apparatus protein (NuMA), an abundant component of NMP in freshly voided spot urine. This painless noninvasive assay provides absolute positive or negative results like pregnancy test within 30 minutes without having any dependency upon intact cancer cells, expert cytopathologist and dedicated laboratory. Low cost, rapid results and simplicity of execution lead to its FDA approval as the only in-office test for the diagnosis of BC.^10^

Although NMP-22 is considered an effective alternative alone or in combination with urine cytology in the diagnosis, screening and surveillance of BC, the clinical evidence generated by several randomized, double-blinded trials conducted recently revealed inconsistent, fluctuating results regarding its diagnostic accuracy.^8^ Moreover, to our knowledge very limited local data was found addressing this aspect leading to weak endorsement of use of this potentially-effective diagnostic modality in our setup.^11^ We aimed to evaluate the diagnostic accuracy of NMP-22 and voided urine cytology in the detection of TCC in our target population.

## METHODS

This cross sectional validation study was conducted at Armed Forces Institute of Urology (AFIU) Rawalpindi, Pakistan form July 2018 to July 2019 after approval of the study protocol from the hospital ethical review board (ERB). Non probability consecutive sampling technique was used to enroll 380 patients after satisfying inclusion criteria (all patients having age 18-75 years of either gender with provisional diagnosis of TCC bladder who were planned to undergo Cystoscopy) while patients already diagnosed with TCC bladder or upper urinary tract, renal malignancy or on dialysis, active gross hematuria, recent history (within two weeks) of urethral instrumentation / catheterization, bladder stones or having active urinary tract infection were excluded. Written informed consent was obtained and demographic details (name, age, and gender) noted.

Voided midstream urine (MSU) was collected at outpatient visit and prior to any treatment, according to the standard protocol for urine collection defined by the Human Kidney and Urine Proteome Project (HKUPP) network.^12^ Collected urine was divided into two aliquots; one underwent NMP22 analysis (Qualitative ALERE™ NMP22® BladderChek®)^13^ at urology laboratory AFIU according to manufacturers ‘protocol while other portion sent to cytopathology laboratory AFIP, Rawalpindi.

Both air dried and wet fixed slides (ThinPrep slides Cytyc Corporation, Marlborough, MA) were prepared by method of direct smearing and cyto-centrifuge. After samples were centrifuged at 2000 rpm for five minutes the supernatant was removed to produce cell pellets which were washed with Cytolyt® Solution. Two to three drops of each patient sample was transferred into PreservCyt® Solution and fixed for 15 minutes. Air dried slides were stained with Diff-Quik® stain (MICROPTIC S.L. Barcelona Spain) while Papanicolaou stain used for wet fixed slides. A consultant cytopathologist evaluated all specimens according to Paris classification system for reporting urine cytology.^14^ While classes 1 and 2 were considered negative, classes 4 and 5 were deemed positive for bladder cancer, all patients with class 3 (atypical urothelial cells) cytological findings but negative cystoscopy were excluded. Presence or absence of urothelial bladder cancer was confirmed with rigid cystoscopy under spinal / general anesthesia conducted by consultant urologist having at least 10 years of experience assisted by researcher. All this information was recorded on a specially designed proforma by researcher himself.

### Statistical analysis

It was performed using IBM SPSS Statistics for Windows, Version 24.0. (Armonk, NY: IBM Corp). Descriptive statistics were used to calculate means ± standard deviation (SD) for quantitative variables i.e. age. The qualitative variable i.e., gender and TCC (on NMP22, urine cytology and cystoscopy) were presented as frequency and percentage and chi square test applied to determine significance. Contingency tables were generated to calculate sensitivity, specificity, positive predictive value (PPV), negative predictive value (NPV) and diagnostic accuracy of NMP22, urine cytology and combined NMP 22 / urine cytology taking cystoscopy as gold standard. Receiver operating characteristic (ROC) curve analysis performed and the area under the curve (AUC) compared among these tests. Effect modifiers like age and gender were controlled by stratification. McNemar’s test used to compare sensitivity and specificity (P-value ≤0.05 was considered significant).

## RESULTS

**Fig.1:**
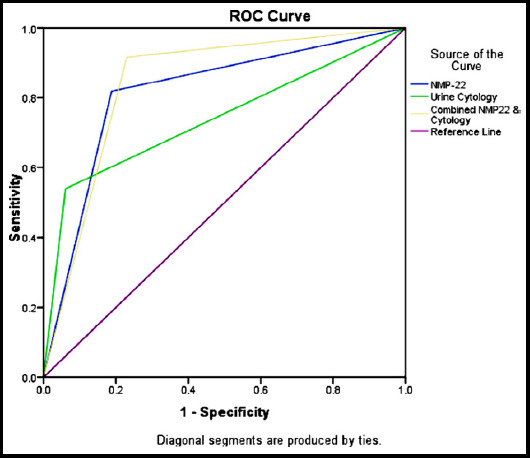
The ROC curve and AUC analysis of urine cytology, NMP 22 and combined NMP22 / cytology in diagnosis of BC.

The study analyses included 380 patients. The average age of patients was 53.08± 12.41 years with age range (24-75) years and male to female ratio 3.75:1(300 males vs. 80 females). Rigid cystoscopy revealed BC in 215 (56.6%) patients while 165 (43.4%) were found negative. NMP 22 test showed BC in 207(54.5%) patients and 173(45.5%) had no cancer. Urine cytology was positive for TCC in 126 (33.2%) and negative in 254 (66.8%). In comparison to gold standard rigid cystoscopy, urine cytology revealed sensitivity 54% and specificity 93.9% (statistically significant p-value <0.001), NMP 22 had sensitivity 81.9% and specificity 81.2% (statistically significant p-value <0.001) while combination of both tests showed sensitivity 91.6% and specificity 77% (statistically significant p-value <0.001) (Table-I). Combination of NMP 22 / cytology outperformed NMP 22 and urine cytology in terms of sensitivity (91.63 vs 81.83 vs 53.96), NPV (87.59 vs 77.46 vs 61.02) and diagnostic accuracy (85.26 vs 81.58 vs 71.32) but at the cost of specificity (76.97 vs 81.21 vs 93.94) and PPV (83.83 vs 85.02 vs 92.06). Urine cytology revealed highest specificity (93.94%) and PPV (92.06%) while NMP 22 showed good balance of sensitivity, specificity, PPV, NPV and diagnostic accuracy (81.86% (76.05- 86.77), 81.21% (74.40-86.86), 85.02% (80.43- 88.69), 77.46% (71.93- 82.17), 81.58% (77.31- 85.35 respectively) (Table-II). Data was stratified according to age and sex for all three tests. Cytology performed equally in all age groups and both gender while NMP 22 had improved sensitivity and NPV in patients > 45 years of age. Combination of both performed better in female sub group (Table-III). ROC curve analysis revealed combination to have highest AUC (0.843 vs 0.815 vs .73) as compared to NMP 22 and Cytology, the difference being statistically significant (p-value <0.001) (Fig.1).

**Table-I:** Diagnostic performance of Urine cytology, NMP 22 and combination against gold standard rigid cystoscopy (n=380).

Diagnostic test	Sensitivity	Specificity
NMP 22 vs Urine Cytology	<0.001	<0.001
Combination Vs Urine Cytology	<0.001	<0.001
Combination vs NMP 22	<0.001	0.016

**Table-II:** Comparison of the diagnostic accuracy of NMP-22, voided urine cytology and combined NMP-22 & Cytology in diagnosis of bladder cancer keeping rigid cystoscopy gold standard (n=380).

Test	TP	TN	FP	FN	Sensitivity (95% CI)	Specificity (95% CI)	PPV (95% CI)	NPV (95% CI)	Accuracy (95% CI)
Cytology	116	155	10	99	53.95% (47.04-60.75)	93.94% (89.14- 97.06)	92.06% (86.27-95.54)	61.02% (57.41- 64.52)	71.32% (66.48- 75.81)
NMP-22	176	134	31	39	81.86% (76.05- 86.77)	81.21% (74.40-86.86)	85.02% (80.43- 88.69)	77.46% (71.93- 82.17)	81.58% (77.31- 85.35)
Combined	197	127	38	18	91.63% (87.09- 94.96)	76.97% (69.79- 83.16)	83.83% (79.64- 87.30)	87.59% (81.81-91.71)	85.26% (81.29-88.67)

**Table-III:** Data stratification of sensitivity, specificity, PPV, NPV and accuracy of urine cytology, NMP-22 and Combined NMP-22 & Cytology with respect to age and gender (n=380).

Test	TP	TN	FP	FN	Sensitivity (95% CI)	Specificity (95% CI)	PPV (95% CI)	NPV (95% CI)	Accuracy (95% CI)
Cytology Age									
<45 Years	30	45	04	24	55.56% (41.4-69.08)	91.84% (80.4- 97.73)	88.24% (74- 95.18)	65.22% (57.91-71.88)	72.82% (63.16-81.12)
>45 Years	86	110	06	75	53.42% (45.4- 61.3)	94.83% (89.08- 98.08)	93.48% (86.65-96.94)	59.46% (55.29- 63.5)	70.76% (65.02- 76.05)
Gender									
Male	93	122	08	77	54.71% (46.9- 62.34)	93.85% (88.23- 97.31)	92.08% (85.42-5.84)	61.31% (57.18- 65.28)	71.67% (66.2- 76.7)
Female	23	33	02	22	51.11% (35.77- 66.3)	94.29% (80.84- 99.3)	92% (74.39-97.85)	60% (52.39- 67.15)	70% (58.72- 79.74)
NMP-22 Age									
< 45 Years	43	39	10	11	79.63% (66.47- 89.37)	79.59% (65.66- 89.76)	81.13% (70.88-88.37)	78% (67.25- 85.96)	79.61% (70.54- 86.91)
>45 Years	133	95	21	28	82.61% (75.86- 88.12)	81.9% (73.67- 88.43)	86.36% (81.04-90.37)	77.24% (70.56- 82.7)	82.31% (77.3- 86.62)
Gender									
Male	139	106	24	31	81.76% (75.13- 87.26)	81.54% (73.79- 87.8)	85.28% (80.03-89.33)	77.37% (71.11- 82.61)	81.67% (76.82- 85.88)
Female	37	28	07	08	82.22% (67.95% to 92.0)	80% (63.06- 91.56)	84.09% (72.88-91.22)	77.78% (64.63- 87.02)	81.25% (70.97- 89.11)
Combined Age									
<45 years	51	37	12	03	94.44% (84.6- 98.84)	75.51% (61.13- 86.66)	80.95% (72.13- 87.47)	92.5% (80.24- 97.4)	85.44% (77.12- 91.6)
>45 Years	146	90	26	15	90.68% (85.1- 94.69)	77.59% (68.91- 84.8)	84.88% (79.95- 88.77)	85.71% (78.58-90.75)	85.20% (80.46- 89.16)
Gender									
Male	155	100	30	15	91.18% (85.86- 94.98)	76.92% (68.72- 83.86)	83.78% (79- 87.65)	86.96% (80.29- 91.60)	85% (80.45- 88.84)
Female	42	27	08	03	93.33% (81.73-98.6)	77.14% (59.86- 89.58)	84% (73.97- 90.65)	90% (74.82- 96.46)	86.25% (76.73-92.93)

## DISCUSSION

BC is highly common lethal disease affecting elderly posing very high financial and administrative burden on health care systems across the world.^15^ Rigid cystoscopies is criterion standard for diagnosis as well as surveillance nonetheless it is invasive, expensive and resource intensive. Urine cytology is another commonly used gold standard modality having excellent specificity but very low sensitivity especially for low grade tumors, requirement of trained cytopathologist and paucity of objective interpretation criteria.^16^ These compelling limitations prompted approval of several noninvasive biomarkers though with conflicting diagnostic accuracy quoted for primary and recurrent BC. The NMP-22 BladderChek test is an in vitro enzyme immunoassay approved by FDA for in-office testing of BC and harbors potentials to revolutionize BC diagnostic and surveillance strategies.^17^ In this prospective validational study we investigated the diagnostic value of urinary NMP 22 against urine cytology for BC.

Our data showed concordance with already published international data on the subject albeit with different study population. Stamfer et al.^18^ evaluated 231 patients with a history of superficial TCC of the bladder and found that NMP22 was two times more sensitive than cytology while Yafi FA et al.^19^, Dogan C et al.^20^ demonstrated sensitivity of 70-80% for NMP22 test. In comparison, cytology showed sensitivity of ∼10–40%. Our results revealed significantly higher sensitivity of NMP-22 than urine cytology (81.58 vs 53.96% p value <0.001) while cytology was found more specific (81.83% vs 93.94%).

**Table:** Area under the Curve

Test Result Variable(s)	Area	Std. Error	Asymptotic Sig	Asymptotic 95% Confidence Interval	

				Lower Bound	Upper Bound
Urine Cytology	0.739	0.025	0.000	0.690	0.789
NMP-22	0.815	0.023	0.000	0.770	0.861
Combined NMP22 / Cytology	0.843	0.022	0.000	0.799	0.887

A study conducted by Pichler R et al.^21^ compared NMP-22 and urinary bladder cancer antigen (UBC) rapid to urinary cytology. Analysis of 75 patients showed sensitivity and specificity both NMP-22 and urinary cytology comparable (12.9% & 100% vs 25.8% & 100% respectively), the sensitivity both tests quite less as compared to our study while specificity comparable to our results. AUC for both was also quite less as compared to our data (0.63 cytology & 0.56 NMP-22 vs 0.73 cytology & 0.82 NMP-22). In another study published BJUI in 2018, Pichler R et al.^22^ compared Xpert Bladder Cancer (BC) Monitor with cystoscopy and urinary cytology. The sensitivity, NPV and AUC of cytology was 0.33, 0.76 and 0.547. NPV value in our study for cytology was 0.6 while AUC was 0.730. The sensitivity, specificity and NPV of cytology depicted by Zhou L et al.^23^ are comparable to our results (30%, 91% & 86%). Another study conducted by Bier S. et al.^24^ included 758 bladders and 385 upper urinary tract (UUT) samples, the sensitivity of cytology & NMP 22 were found 74.6 & 100% for UUT and 59.3 & 62.5% for bladder samples while specificity was 66.6 & 50.9% for UUT and 82.9 & 31.3 for bladder samples.

However, the results published by Lotan Y. et al.^25^ contrary to our data. The sensitivity of NMP-22 BladderChek was 0.11 as compared to 0.82 while NPV was 0.86 (0.77 in our study). These differing results could be attributed to the different thresholds used in these studies and differences in the number of patients in each series. Our analytical results depicted superiority of NMP-22 to all other tests when all parameters of sensitivity, specificity, PPV, NPV and accuracy are taken into account together, a finding reported by Goodison S et al.^26^ as well.

Todenhofer T et al.^27^ studied diagnostic accuracy of combination of various markers with urine cytology. Their results showed very good performance for combination of cytology with FISH, uCyt+ but no additional affect by combining with NMP 22. However, the combination of both tests revealed a trade of significantly better sensitivity, NPV and accuracy at cost of low specificity and PPV in our study. To add further combination can act as very good screening tool owing to high sensitivity although in the wake of increasing cost and missing 9% of the cases. Although the combination of the NMP22 test and cytology cannot replace cystoscopy in the surveillance protocol at present, it can potentially reduce frequency of cystoscopic follow up reducing burden on patient as well as health care provider.

### Limitations of the study

The results of present study should be interpreted with care as it’s a single center study, grade, stage as well as upper tract tumor were not taken into account leading to potential bias in interpretation of results. Moreover, cytopathologist involved were more than one which may have caused inter-observer variability.

## CONCLUSIONS

NMP22 is a quick, point of care test having higher sensitivity, NPV and accuracy but similar specificity and PPV to urine cytology for detection of TCC urinary bladder. Combination outperformed both in terms of sensitivity while having modest specificity. At present these noninvasive markers can’t be recommended to replace cystoscopy however the NMP22 alone or in combination provides new avenues to adjunct cystoscopy in the diagnosis, screening and surveillance of BC.

### Author`s Contribution

**MTS and MRZ** conceived, designed and did statistical analysis & editing of manuscript.

**MTS, HU and SU** did data collection.

**MRZ, HA, ZIM and KS** reviewed, provided technical support and final approval of manuscript.

All authors done critical revision of the manuscript for important intellectual content.

All authors of this paper have equally contributed to this study, approved the final version to be published and are responsible for integrity of research.
